# Characterization of Adult Rat Astrocyte Cultures

**DOI:** 10.1371/journal.pone.0060282

**Published:** 2013-03-28

**Authors:** Débora Guerini Souza, Bruna Bellaver, Diogo Onofre Souza, André Quincozes-Santos

**Affiliations:** Department of Biochemistry, Institute of Basic Health Sciences, Federal University of Rio Grande do Sul, Porto Alegre, RS, Brazil; Albany Medical College, United States of America

## Abstract

Astrocytes, a major class of glial cells, regulate neurotransmitter systems, synaptic processing, ion homeostasis, antioxidant defenses and energy metabolism. Astrocyte cultures derived from rodent brains have been extensively used to characterize astrocytes' biochemical, pharmacological and morphological properties. The aims of this study were to develop a protocol for routine preparation and to characterize a primary astrocyte culture from the brains of adult (90 days old) Wistar rats. For this we used enzymatic digestion (trypsin and papain) and mechanical dissociation. Medium exchange occurred from 24 h after obtaining a culture and after, twice a week up to reach the confluence (around the 4^th^ to 5^th^ week). Under basal conditions, adult astrocytes presented a polygonal to fusiform and flat morphology. Furthermore, approximately 95% the cells were positive for the main glial markers, including GFAP, glutamate transporters, glutamine synthetase and S100B. Moreover, the astrocytes were able to take up glucose and glutamate. Adult astrocytes were also able to respond to acute H_2_O_2_ exposure, which led to an increase in reactive oxygen species (ROS) levels and a decrease in glutamate uptake. The antioxidant compound resveratrol was able to protect adult astrocytes from oxidative damage. A response of adult astrocytes to an inflammatory stimulus with LPS was also observed. Changes in the actin cytoskeleton were induced in stimulated astrocytes, most likely by a mechanism dependent on MAPK and Rho A signaling pathways. Taken together, these findings indicate that the culture model described in this study exhibits the biochemical and physiological properties of astrocytes and may be useful for elucidating the mechanisms related to the adult brain, exploring changes between neonatal and adult astrocytes, as well as investigating compounds involved in cytotoxicity and cytoprotection.

## Introduction

Astrocytes are key cells in the central nervous system (CNS) involved in the maintenance of the extracellular environment and in the stabilization of cell-cell communications under physiological and pathological conditions. [1], [2], [3], [4], [5]. Specifically, astrocytes regulate neurotransmitter systems; ionic homeostasis; the metabolic support of neurons; energy metabolism; synaptic information processing, plasticity and neuronal excitability; detoxification; defense against oxidative stress; metal sequestration; maintenance of the blood-brain barrier; guidance of neuronal migration; and immune function [5], [6], [7]. In addition, these versatile cells are the most resilient cells in the CNS [7]. Astrocytes are also responsible for clearing extracellular glutamate, the main neurotransmitter of excitatory signaling in the mammalian CNS, due to their high-affinity glutamate transporters: GLAST (EAAT1) and GLT1 (EAAT2) [8], [9], [10], [11], [12], [13], [14]. Once taken up by astrocytes, glutamate is converted into glutamine by glutamine synthetase (GS – EC 6.3.1.2) [15], [16], [17], [18]. Glutamate uptake is also important for maintaining the levels of glutathione (GSH), a major antioxidant molecule in the brain [15], [16], [19], [20].

Classically, astrocyte cultures are obtained from the perinatal rodent brain and have been extensively used to characterize the biochemical, pharmacological and morphological properties of the CNS [21], [22], [23]. Primary cultures of astrocytes express important markers, such as GFAP (glial fibrillary acidic protein), S100B and GS proteins [24], [25], [26], [27], [28], [29]. In addition, astrocyte cultures are extremely useful for metabolic studies of the glutamatergic system, oxidative stress and the inflammatory response [3], [11], [30], [31]. However, cultures of astrocytes from the adult brains are poorly studied and could represent an important tool for understanding glial functions. Adult astrocytes contain well-established connections and are more organized than newborn tissue, which is plastic and labile to stimuli [32], [33], [34]. Thus, astrocyte cultures obtained from adult rats may respond more reliably and help to elucidate the role of astrocytes in *in vivo* processes of brain aging.

The aim of this study was to establish an *in vitro* culture model of astrocytes obtained from adult (90 days old) Wistar rats and to evaluate the astrocytes' functions. First, we determined the levels of GFAP, GS, S100B, vimentin and aldehyde dehydrogenase family 1, member L1 (ALDH1L1 – EC 1.5.1.6). Further, we evaluated glutamate uptake, GS activity, GSH intracellular levels and glucose uptake. Finally, we investigated the response of the cell cultures to external stimuli, such as oxidants, antioxidants and inflammatory conditions, by measuring the levels of intracellular reactive oxygen species (ROS), glutamate parameters, cytoskeletal signaling pathways and the inflammatory response.

## Materials and Methods

### Reagents

Dulbecco's Modified Eagle's Medium/F12 and other materials for cells culture were purchased from Gibco. Papain and H_2_O_2_ were from Merck. DNase, cysteine, albumin, ZnCl_2_, polyclonal anti-β-tubulin III, polyclonal anti-Glutamine synthetase, monoclonal anti-S100B, monoclonal anti-vimentin, o-phtaldyaldehyde, lysophosphatidic acid (LPA), 2′-7′-dichorofluorescein diacetate (DCFHDA), propidium iodide (PI), γ-glutamylhydroxamate, MTT Formazan and resveratrol were from Sigma-Aldrich. SB203580 and 4′,6′-diamino-2-phenylindole (DAPI) were from Calbiochem. Polyclonal anti-GFAP was from Dako; monoclonal anti-NeuN was from Millipore; monoclonal anti-CD11 was from Invitrogen; polyclonal anti-GLAST and anti-GLT1 were from Alpha Diagnostic; monoclonal anti-Aldehyde dehydrogenase family 1 member L1 was from Neuromab; monoclonal anti-GAPDH was from Chemicon. L-[^3^H]-glutamate, 2-Deoxy-D-[1,2-^3^H]glucose ([^3^H]2DG), nitrocellulose membrane and ECL kit were from Amersham. Alexa Fluor® 488 (A_max_ = 493; E_max_ = 519) or 594 (A_max_ = 591; E_max_ = 614)-conjugated AffiniPure antibodies were from Jackson ImmunoResearch. All other chemicals were from common commercial suppliers.

### Animals

Male Wistar rats (90 days old) were obtained from our breeding colony (Department of Biochemistry, UFRGS, Brazil), maintained under controlled environment (12 h light/12 h dark cycle; 22±1°C; *ad libitum* access to food and water). All animal experiments were performed in accordance with the NIH Guide for the Care and Use of Laboratory Animals and were approved by the Federal University of Rio Grande do Sul Animal Care and Use Committee (process number 21215).

### Cell cultures

#### Primary astrocyte cultures from adult rats

Male Wistar rats (90 days old) had their cerebral cortices aseptically dissected and meninges removed. During the dissection, the cortices were kept in HBSS (Hank's Balanced Salt Solution) containing 0.05% trypsin and 0.003% DNase and were kept at 37°C for 15 min. The tissue was then mechanically dissociated for 15 min using a Pasteur pipette and centrifuged at 400 g for 5 min. The pellet was resuspended in a solution of HBSS containing 40 U papain/ml, 0.02% cysteine and 0.003% DNase and again gently mechanically dissociated for 15 min with a Pasteur pipette. After another centrifugation step (400 g, 5 min), the cells were resuspended in HBSS containing only DNase (0.003%) and left for decantation for 30–40 min. The supernatant was collected and centrifuged for 7 min (400 g). The cells from supernatant were resuspended in DMEM/F12 [10% fetal bovine serum (FBS), 15 mM HEPES, 14.3 mM NaHCO_3_, 1% fungizone and 0.04% gentamicin], plated in 6- or 24-well plates pre-coated with poly-L-lysine and cultured at 37°C in a 95% air/5% CO_2_ incubator. The cells were seeded at 3–5×10^5^ cells/cm^2^. The number of cells obtained by this method from one cortex of adult Wistar rat is approximately 15×10^6^ cells.

#### Cell culture maintenance

The first medium exchange occurred 24 h after obtaining a culture. During the 1^st^ week, the medium change occurred once every two days and, from the 2^nd^ week on, once every four days. From the 3^rd^ week on, the cells received medium supplemented with 20% FBS. Around the 4^th^ to 5^th^ week, the cells reached the confluence and were used for the experiments. No dibutyryl cAMP was added to the culture medium.

#### Treatments

After the characterization of the astrocyte cultures from adult rats, we combined biochemical and morphological tools to explore the responses of cells to different stimuli. The cells were exposed to H_2_O_2_ (50 and 100 µM) for 1, 3 or 6 h to evaluate the oxidative and inflammatory responses. The effect of H_2_O_2_ on cytoskeleton and the activation of p38 MAPK (through a specific inhibitor - SB203580) were also examined. In another set of experiments, we evaluated the protective effects of resveratrol against H_2_O_2_ exposure. The cells were pre-treated with 100 µM resveratrol for 1 h before the H_2_O_2_ treatment (50 µM for 3 h). Glutamate exposure stimulates glycolysis, i.e., increase in glucose uptake in astrocytes [35]. Thus, we also examined whether astrocytes are able to take up glucose after treatment with 500 µM glutamate for 20 min. It has been shown that Zinc is a neurotoxin [36]. The Zinc toxicity on cytoskeleton and the possible involvement of Rho A signaling pathway in its effects were also assessed. Cells were incubated with 50 µM ZnCl_2_ for 3 h in the presence or absence of 2 µM lysophosphatidic acid (LPA), which was added 30 min before the treatment. Inflammatory responses were also measured by exposing the cells to lipopolysaccharides (LPS), 10 µg/ml for 3 h. During all incubations, the cells were maintained at 37°C in an atmosphere of 5% CO_2_/95% air.

### Immunocytochemistry

Immunocytochemistry was performed as described previously by our group [37]. Briefly, cell cultures were fixed with 4% paraformaldehyde for 20 min and permeabilized with 0.1% Triton X-100 in PBS for 5 min at room temperature. After blocking overnight with 4% albumin, the cells were incubated overnight with anti-GFAP (1∶400), anti-glutamine synthetase (1∶10,000), anti-β-tubulin III (1∶500), anti-NeuN (1∶50) or anti-CD11 (1∶400) at 4°C, followed by PBS washes and incubation with a specific secondary antibody conjugated with Alexa Fluor® 488 (green staining) or 594 (red staining) for 1 h at room temperature. For all the immunostaining-negative controls, the reactions were performed by omitting the primary antibody. No reactivity was observed when the primary antibody was excluded. Cell nuclei were stained with 0.2 µg/ml of 4′,6′-diamidino-2-phenylindole (DAPI). The cells were visualized with a Nikon inverted microscope and the images were transferred to a computer with a digital camera (Sound Vision Inc.).

### Western blot analyses

The presence of specific proteins was evaluated by Western blot analyses. Briefly, cells were removed from plates after reaching confluence using lysis solution with 4% SDS, 2 mM EDTA, 50 mM Tris-HCl (pH 6.8). Protein content was measured, the samples were standardized in sample buffer [62.5 mM Tris-HCl (pH 6.8), 4% (v/v) glycerol, 0.002% (w/v) bromophenol blue] and boiled at 95°C for 5 min. From the male Wistar rats (90 days old) used for primary astrocyte cultures, we separated a part of the cortex (rat cortex homogenate) and it was used as a positive control for Western blot analyses. The rat cortex homogenate presents the expression of specific proteins, such as GFAP, vimentin, GS, ALDH1L1, S100B, GLAST and GLT1. The samples were then loaded into the gels (50 µg protein) together rat cortex homogenate, submitted to electrophoresis in a SDS-polyacrylamide gel and blotted onto nitrocellulose membranes. Adequate loading of each sample was confirmed using Ponceau S staining. After blocking with 4% albumin for 2 h, the membranes were incubated overnight with one of the following antibodies: anti-vimentin (1∶400), anti-S100B (1∶2,000), anti-GFAP (1∶1,000), anti-glutamine synthetase (1∶20,000), anti-GLT1 (1∶2,000), anti-GLAST (1∶2,000), anti-ALDH1L1 (1∶1,000), anti-GAPDH (1∶300) or anti-β-tubulin III (1∶1500). After incubating overnight with the primary antibody (4°C), the membranes were washed and incubated with a peroxidase-conjugated anti-rabbit immunoglobulin (IgG) (in the case of anti -GLT1, -GLAST, -GS, -β tubulin III and –GFAP) or with peroxidase-conjugated anti-mouse immunoglobulin (in the case of anti -ALDH1L1, - vimentin, -GAPDH, -S100B) at a dilution of 1∶2,000 for 1 h. Chemiluminescence signals were detected using an ECL kit and Kodak film.

### Glutamate uptake

After the cells reached confluence, the glutamate uptake was performed as previously described [38] with some modifications. Briefly, astrocytes were incubated at 37°C in HBSS containing the following components (in mM): 137 NaCl, 5.36 KCl, 1.26 CaCl_2_, 0.41 MgSO_4_, 0.49 MgCl_2_, 0.63 Na_2_HPO_4_, 0.44 KH_2_PO_4_, 4.17 NaHCO_3_ and 5.6 glucose, adjusted to pH 7.4. The assay was started by the addition of 0.1 mM L-glutamate and 0.33 µCi/ml L-[2,3-^3^H] glutamate. The incubation was stopped after 7 min by removal of the medium and rinsing the cells twice with ice-cold HBSS. The cells were then lysed in a solution containing 0.5 M NaOH. Incorporated radioactivity was measured in a scintillation counter. Sodium-independent uptake was determined using N-methyl-D-glucamine instead of sodium chloride. Sodium-dependent glutamate uptake, considered “specific uptake”, was obtained by subtracting the sodium-independent uptake from the total uptake.

### Glutamine synthetase activity

After the cells reached confluence, the enzymatic assay was performed as previously described [39]. Briefly, cell homogenate (0.1 ml – about 50 µg) was added to 0.1 ml of the reaction mixture containing (in mM): 10 MgCl_2_, 50 L-glutamate, 100 imidazole-HCl buffer (pH 7.4), 10 2-mercaptoethanol, 50 hydroxylamine-HCl and 10 ATP, and incubated for 15 min (37°C). The reaction was stopped by the addition of 0.4 ml of a solution containing (in mM): 370 ferric chloride, 670 HCl, and 200 trichloroacetic acid. After centrifugation, the absorbance of the supernatant was measured at 530 nm and compared to the absorbance generated using standard quantities of γ-glutamylhydroxamate treated with a ferric chloride reagent.

### Glutathione content

After the cells reached confluence, GSH levels were assessed as previously described [40]. Astrocyte homogenates (by about 50 µg) were diluted in 100 mM sodium phosphate buffer (pH 8.0) containing 5 mM EDTA, and the protein was precipitated with 1.7% meta-phosphoric acid. The supernatant was assayed with o-phthaldialdehyde (1 mg/ml methanol) at room temperature for 15 min. Fluorescence was measured using excitation and emission wavelengths of 350 and 420 nm, respectively. A calibration curve was performed with standard GSH solutions (0–500 µM). GSH concentrations were calculated as nmol/mg protein.

### 2-Deoxy-D-[1,2-^3^H]glucose ([^3^H]2DG) uptake

After cells reached confluence, basal glucose uptake and glutamate-stimulated glucose uptake were assessed as previously described [35]. Briefly, for basal uptake, the cells were rinsed once with HBSS and incubated with DMEM/F12 1%FBS containing 1 µCi/ml [^3^H]2DG for 20 min at 37°C. For glutamate-stimulated glucose uptake, the cells were rinsed once with HBSS and incubated with DMEM/F12 1%FBS for 2 h at 37°C. The medium was then replaced by DMEM/F12 supplemented with 1%FBS containing 1 µCi/ml [^3^H]2DG, in the presence or not of 500 µM glutamate for 20 min at 37°C. After incubations, the cells were rinsed with HBSS and lysed overnight with NaOH 0.3 M. Incorporated radioactivity was measured in a scintillation counter. Cytochalasin B (10 µM) was used as a specific glucose transporter inhibitor. Glucose uptake was determined by subtracting uptake with cytochalasin B from total uptake.

### Cell morphology and cell viability

Morphological studies were performed using phase contrast optics. Membrane integrity was assessed by fluorescent image analysis (Nikon inverted microscope using a TE-FM Epi-Fluorescence accessory) of propidium iodide (PI) uptake (at 7.5 µM) [39] at 37°C in an atmosphere of 5% CO_2_/95% air in DMEM/F12 supplemented with 5% FBS. Cell viability was determined using a MTT Formazan assay (activity of mitochondrial dehydrogenases). Briefly, 0.05 mg/ml of MTT was added to the incubation medium. After 3 hours of incubation, the medium from each well was gently removed by aspiration and dimethylsulfoxide (DMSO) was added to each well followed by incubation and shaking for 5 min. The formazan product generated during the incubation was solubilized in DMSO and measured at 560 and 650 nm. Only viable cells are able to reduce MTT.

### Intracellular ROS levels

Intracellular ROS production was detected using the nonfluorescent cell permeating compound, 2′-7′-dichlorofluorescein diacetate (DCFHDA) on cells under basal conditions or treated with H_2_O_2_ (50 µM). DCFHDA is hydrolyzed by intracellular esterases to dichlorofluorescin (DCFH), which is trapped within the cell. This nonfluorescent molecule is then oxidized to fluorescent dichlorofluorescin (DCF) by the action of cellular oxidants. Astrocytes were treated with DCFHDA (10 µM) for 30 min at 37°C. Following DCFHDA exposure, the cells were scraped into PBS supplemented with 0.2% Triton X-100. Fluorescence was measured using a plate reader (Spectra Max M5, Molecular Devices) at excitation and emission wavelengths of 485 nm and 520 nm, respectively [37].

### Tumor necrosis factor alpha (TNF-α) measurement

The tumor necrosis factor alpha levels were evaluated in 100 µL of extracellular medium, using a rat TNFα ELISA from PeproTech [41].

### Cytoskeleton analyses – actin labeling

For actin-labeling analyses, the cells were fixed for 20 min with 4% paraformaldehyde in 0.1 M phosphate-buffered saline (PBS), rinsed with PBS, and permeabilized for 10 min in PBS containing 0.2% Triton X-100, which was followed by incubation with 10 µg/ml rhodamine-labeled phalloidin in PBS for 45 min and two washes with PBS. Astrocytes were analyzed and photographed using a Nikon microscope and TE-FM Epi-Fluorescence accessory.

### Protein assay

Protein content was measured using Lowry's method with bovine serum albumin as a standard [42].

### Statistical analyses

Data were statistically analyzed using Student's t-test or a one-way analysis of variance (ANOVA), followed by the Tukey's test. P-values<0.05 were considered significant. All analyses were performed using the Statistical Package for Social Sciences (SPSS) software version 15.0.

## Results

### Glial markers expressed in adult astrocytes

**Figure 1:**
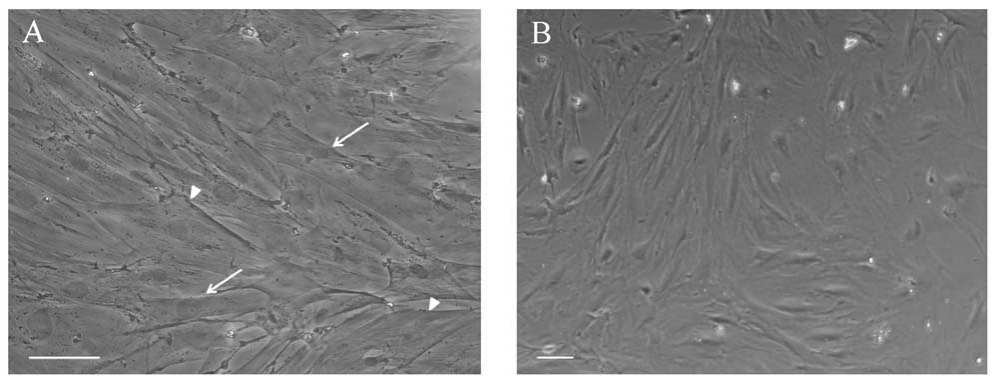
Astrocyte morphology. (**A, B**) Under normal conditions, primary adult astrocytes present a polygonal to fusiform and flat morphology, as shown by phase contrast microscopy. We did not observe morphological alterations during the culture period. The arrowhead indicates a polygonal cell, and the arrow indicates a fusiform cell. Scale bars: (A) = 100 µm, (B) = 50 µm.

Astrocytes possess a specific cytoarchitecture allows them to respond to changes in their microenvironment, which is adequately developed to fulfill their functions [7]. Under basal conditions, adult astrocytes presented a polygonal to fusiform and flat morphology (Fig. 1A, B), as evaluated using phase-contrast microscopy. Moreover, these cells were able to divide until confluence for approximately 4 or 5 weeks (data not shown).

**Figure 2:**
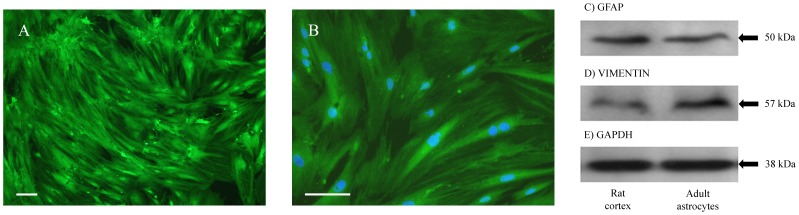
Adult astrocyte cultures present classical astroglial markers. (**A**) Astrocytes show intense immunolabeling for GFAP. (**B**) The cells were counterstained with DAPI (blue). Shown are the merged images only. The data are from 5 independent experiments. Scale bars: (A) = 50 µm, (B) = 100 µm. Representative immunoblot band of rat cortex homogenate and adult astrocytes for (**C**) GFAP, (**D**) vimentin, (**E**) GAPDH. N = 5 for all immunoblot experiments.

Furthermore, the main markers of astrocytes were evaluated. Immunocytochemical analysis demonstrated an intense cytoplasmic immunolabeling of the cytoskeletal protein GFAP, attesting to the astrocytic phenotype of the cultured cells (Fig. 2A, B). The GFAP staining exhibited a meshwork extending across the cytoplasm. Immunoblotting also demonstrated strong GFAP expression compared to rat cortex homogenate (Fig. 2C). Vimentin, another intermediate filament of the cytoskeleton, was also expressed in these cells (Fig. 2D). GAPDH, a protein present at high levels in almost all tissues, was used as a loading control (Fig. 2E).

**Figure 3:**
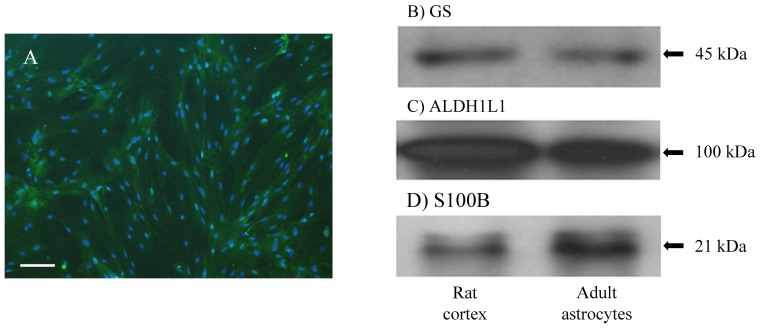
Adult astrocytes cultures express GS, ALDH1L1 and S100B proteins. (**A**) Immunocytochemistry of adult astrocyte cultures for GS with a merge for DAPI staining. Representative immunoblot band for (**B**) GS, (**C**) ALDH1L1 and (**D**) S100B. The left band represents rat cortex homogenate and the right band represents adult astrocytes. N = 5 for all immunoblot experiments.

The GS protein, which is constitutively expressed in astroglial cells, was also evaluated by immunocytochemistry (Fig. 3A) and immunoblotting (Fig. 3B), both of which demonstrated intense GS expression. Recently, it was demonstrated that ALDH1L1, a key enzyme in folate metabolism, may regulate astrocyte division [43]. Immunoblot analysis demonstrated ALDH1L1 expression (Fig. 3C). S100B, a Ca^2+^-binding protein, is abundantly expressed in astrocytes and has been implicated in the regulation of intracellular functions, including proliferation and differentiation. Western blot analysis indicated the presence of the S100B protein (Fig. 3D).

To determine whether the culture contained microglia or neurons after reaching confluence, we used three specific antibodies: (i) anti-CD11b/c (an antibody that recognizes a specific microglial protein), (ii) anti-β-tubulin III (an antibody that recognizes a type of microtubule expressed exclusively in neurons) and (iii) anti-NeuN (an antibody that recognizes a neuronal protein present in neuronal nuclei). Approximately 5% of the cultured cells stained positive for the microglial marker, while neither β-tubulin III nor NeuN were detected (data not shown).

### Adult astrocytes take up glutamate and glucose

Glutamate uptake is essential for maintaining the extracellular glutamate levels below excitotoxic levels [10], [12]. Table 1 depicts the glutamate uptake in adult astrocyte primary cultures. Furthermore, two important glutamate destinations were evaluated by determining the GS activity and the intracellular content of GSH (Table 1). Glutamate uptake also results in the stimulation of glucose utilization [35], and the cultured cells were able to take up glucose.

**Table 1:** Analysis of the main glial parameters in adult astrocytes under basal conditions.

Glial parameter(Basal conditions)	Adult astrocytes
Glutamate uptake(nmol/mg protein/min)	0.9±0.1
GS activity(µmol/mg protein/min)	2.3±0.3
GSH(nmol/mg protein)	18.0±2.0
Glucose Uptake(fmol/mg protein)	240±20

Glutamate uptake, GS activity, GSH intracellular content and glucose uptake were measured after the cells reached confluence. The assays were performed as described in the Materials and Methods section. Data are presented as the mean ± S.E. from 6 experimental determinations performed in triplicate.

**Figure 4:**
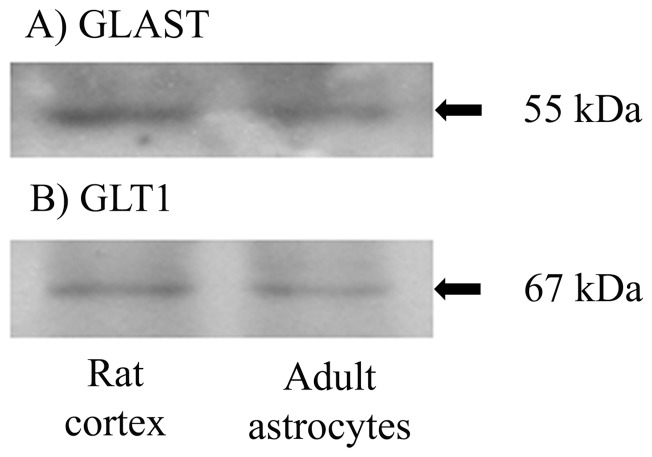
Adult astrocyte cultures express the main glutamate transporters. Representative immunoblot band for (**A**) GLAST and (**B**) GLT1. The left band represents rat cortex homogenate and the right band represents adult astrocytes. N = 5 for all immunoblot experiments.

Moreover, we also detected GLAST and GLT1 expression (Fig. 4A and B, respectively), which are the main glutamate transporter subtypes known to be expressed by mammalian forebrain astrocytes in culture and *in vivo*.

### Astrocytes respond to external stimuli

**Figure 5:**
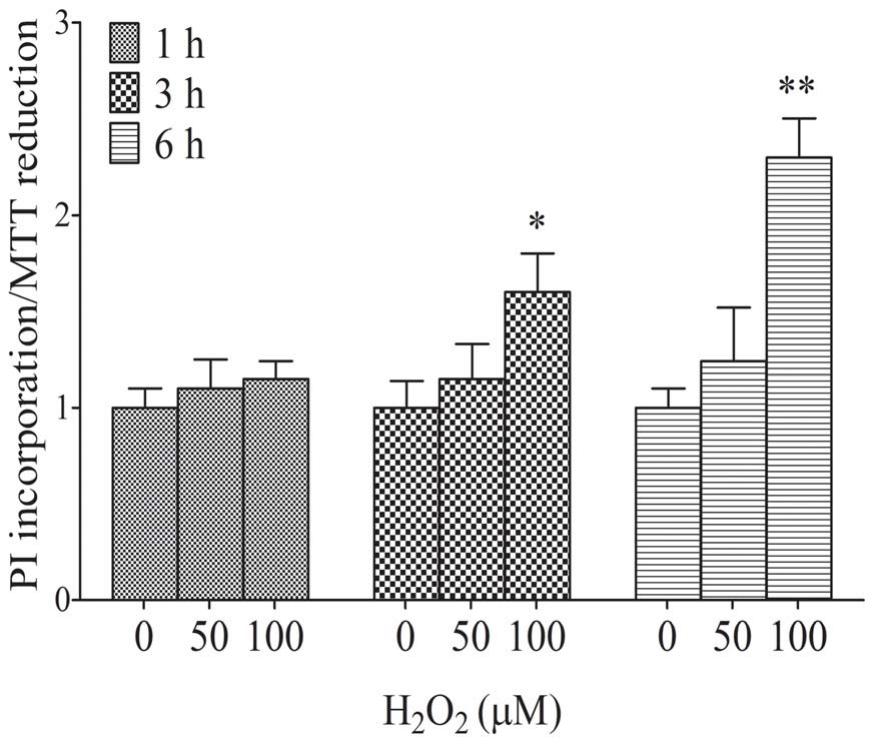
Effects of H_2_O_2_ on adult astrocyte cell viability. Membrane integrity (PI incorporation) and metabolic activity (MTT reduction) were measured as described in the Materials and Methods section. The medium was removed, followed by the addition of DMEM/F12 supplemented with 5% FBS, and the cells were maintained at 5% CO_2_ (37°C) in the presence or absence of H_2_O_2_ for 3 h. Data are expressed as the ratio of PI incorporation to MTT reduction and represent the mean ± S.E. from 4 to 6 experimental determinations performed in triplicate. * P<0.05 and ** P<0.01 indicate significant differences from control conditions.

As glutamate transporters are sensitive to oxidative stress [44], which can impair glutamate uptake, we assessed the response of adult astrocytes to oxidative stress. First, we evaluated the integrity and metabolic activity of cells stimulated with H_2_O_2_ for 1, 3 and 6 h by determining the ratio between PI incorporation and MTT reduction (the higher values indicate reduction in cell viability). After 3 and 6 h of treatment, 100 µM H_2_O_2_ induced an intense reduction in cell viability compared to the control conditions (Fig. 5). The ratio in basal conditions did not change with time. A high concentration of H_2_O_2_ (1 mM) was able to induce cell death.

**Figure 6:**
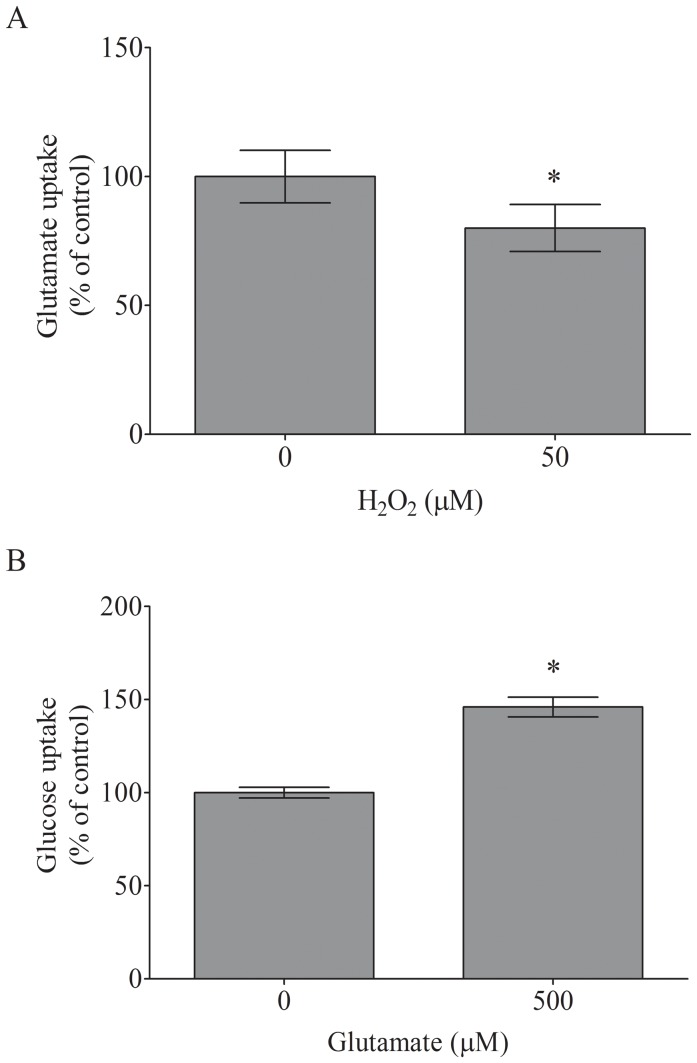
Adult astrocytes take up glutamate (A) and glucose (B). To determine the glutamate uptake, the culture medium was removed followed by the addition of DMEM/F12 supplemented with 5% FBS in the presence or absence 50 µM H_2_O_2_ for 3 h. To determine the glucose uptake, the medium was then replaced by DMEM/F12 supplemented with 1% FBS containing 1 µCi/ml [^3^H]2DG, in the presence or absence of 500 µM glutamate for 20 min. In all procedures, the cells were maintained at 5% CO_2_/37°C. The assays were performed as described in the Materials and Methods section. Absolute values obtained under control conditions were 0.8±0.08 nmol/mg protein/min for glutamate uptake and 172±20 fmol/mg protein for glucose uptake. The data represent the mean ± S.E. from 4 to 6 experimental determinations performed in triplicate. * P<0.05 indicates significant differences from the control values.

Next, we assessed the glutamate uptake under oxidative stress resulting from significant changes in cellular redox status. Figure 6A demonstrates that exposure to 50 µM H_2_O_2_ for 3 h decreased glutamate uptake from 100±12% to 80±13% (P<0.05). We also evaluated the glutamate-induced glucose uptake in adult astrocyte cultures. Treatment with 500 µM glutamate for 20 min increased the glucose uptake from 100±7% to 146±9% (P<0.05, Fig. 6B).

### Resveratrol protects adult astrocytes against oxidative insult induced by H_2_O_2_

**Figure 7:**
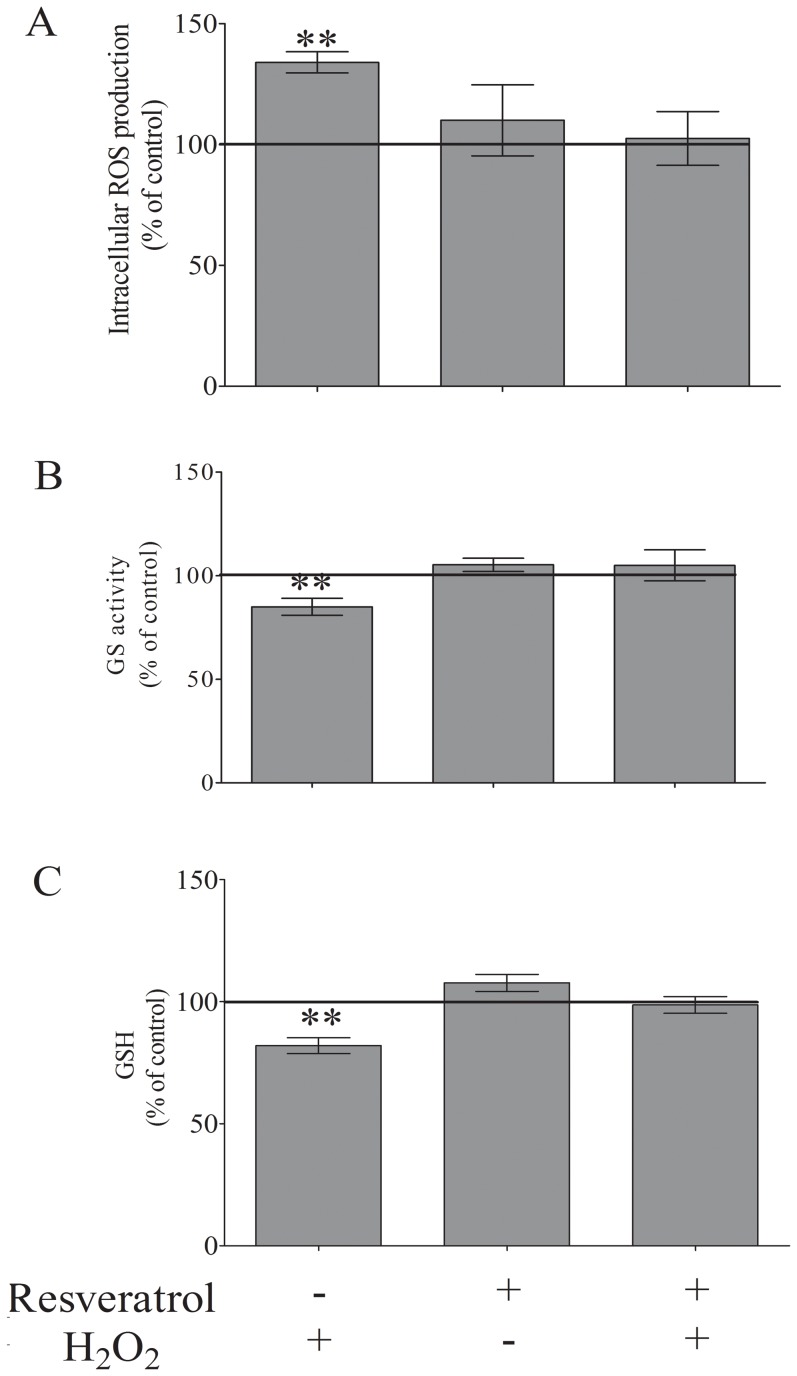
The effect of resveratrol on (A) intracellular ROS production, (B) GS activity and (C) intracellular GSH content during H_2_O_2_-induced oxidative insult. The culture medium was removed followed by the addition of DMEM/F12 supplemented with 5% FBS, and the cells were pre-incubated for 1 h with 100 µM resveratrol followed by the addition of 50 µM H_2_O_2_ for 3 h. Absolute values obtained under control conditions were 2.2±0.6 µmol/mg protein/min for GS activity and 14±1.1 nmol/mg protein for GSH content. The data represent the mean ± S.E. from 4 to 6 experimental determinations performed in triplicate. ** P<0.01 indicates significant differences from the control values.

Our group has previously shown that under oxidative stress, the antioxidant resveratrol modulates important glial functions, such as glutamate uptake, GS activity and GSH levels in astrocyte cultures [29], [37], [45]. Treatment of the culture with 50 µM H_2_O_2_ for 3 h resulted in an increase in ROS levels (34%) and a 15% and 18% reduction in GS activity and GSH intracellular levels, respectively (Fig. 7). Next, the effect of resveratrol against H_2_O_2_-induced oxidative insult was assessed. Pre-treatment with 100 µM resveratrol for 1 h prior to the H_2_O_2_ exposure (50 µM for 3 h) was able to i) prevent the increase in intracellular ROS levels (Fig. 7A) and ii) restore GS activity and GSH content to near-basal levels (Fig. 7B and C, respectively). Resveratrol *per se* did not change these parameters.

### Astrocytic responses to inflammatory stimuli

**Figure 8:**
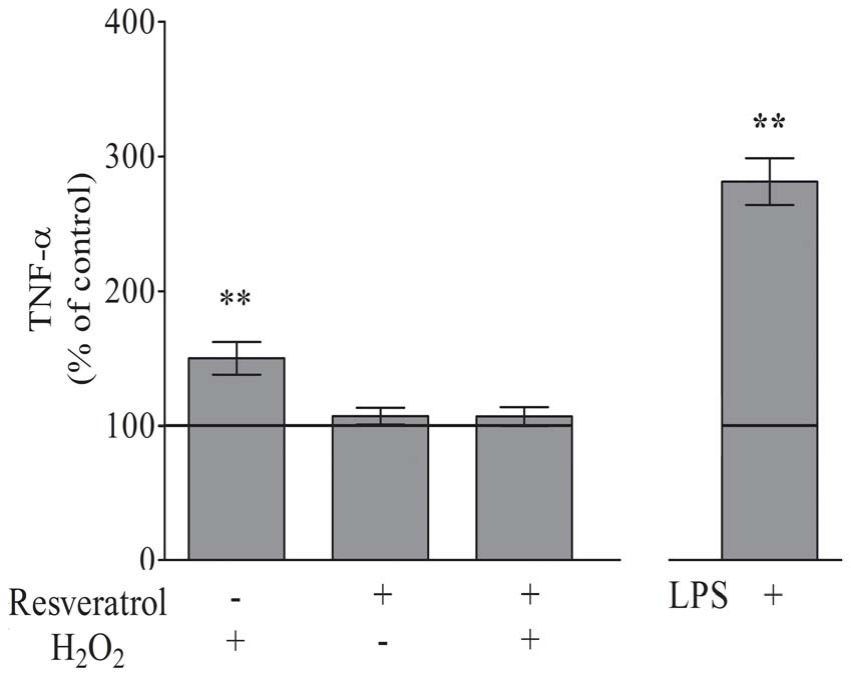
Astrocyte responses to inflammatory stimuli. The culture medium was removed, followed by the addition of DMEM/F12 supplemented with 5% FBS, and the cells were pre-treated with resveratrol (50 µM) for 1 h. After the pre-treatment, H_2_O_2_ (50 µM) was added for 3 h in the presence or absence of resveratrol. Cells were also treated with LPS (10 µg/ml) for 3 h. The data represent the mean ± S.E. from 3 experimental determinations performed in triplicate. ** P<0.01 indicates significant differences from the control value.

ROS play a critical role in inflammatory response [46]. TNF-α is a potent pro-inflammatory cytokine, mainly synthesized by microglia and astrocytes [47]. Adult astrocytes exposure to H_2_O_2_ led to an increase in TNF-α levels by approximately 50% compared to the control conditions (Fig. 8). A 1 h pre-treatment with resveratrol prevented this effect (Fig. 8).

Lipopolysaccharides (LPS) have been widely employed to stimulate inflammatory responses, including in astrocytes [48]. Following the treatment with LPS with LPS (10 µg/ml for 3 h), adult astrocytes displayed an increase in TNF-α levels from 100±8% to 280±12% (P<0.01, Fig. 8).

### H_2_O_2_ and Zn^2+^ induce changes in the astrocyte actin cytoskeleton

**Figure 9:**
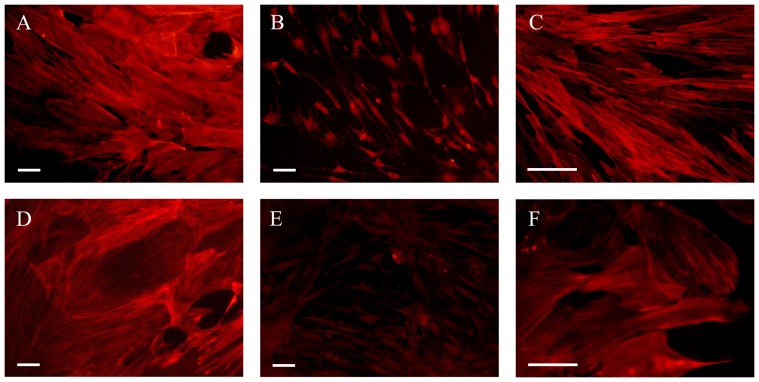
Effects of H_2_O_2_ and Zn^2**+**^ on actin reorganization in adult astrocyte cultures. The culture medium was removed, followed by the addition of DMEM/F12 supplemented with 5% FBS, and the cells were maintained at 5% CO_2_ (37°C) in the presence or absence of H_2_O_2_ or Zn^2+^ for 3 h. Representative images of the rhodamine-phalloidin fluorescence signals of cells exposed to (**A, D**) basal conditions, (**B**) 50 µM H_2_O_2_, (**C**) H_2_O_2_ + SB203580 (5 µM), (**E**) 50 µM Zn^2+^ and (**F**) Zn^2+^ + LPA (2 µM). All images are representative fields from at least 3 experiments performed in duplicate. Scale bars: (A, B, D, E) = 50 µm, (C, F) = 100 µm.

Actin cytoskeleton is the major determinant of the cell morphology, and it is involved in motility, migration and cellular adhesion [49]. Impairment of actin polymerization alters many astrocytic functions, including Ca^2+^ signaling, cell growth and glutamate uptake [50]. In addition, rearrangement of the actin cytoskeleton is an important biological response to several extracellular stimuli, which are frequently mediated by the Rho family of small GTPases [51]. H_2_O_2_ was able to induce cell body retraction and actin reorganization (Fig. 9A, B, C). Zhu et al. [52] have demonstrated that H_2_O_2_ alters the cytoskeleton of newborn astrocytes *in vitro* through activation of the p38 MAPK pathway. Therefore, we used a specific inhibitor of p38 MAPK (SB203580, 5 µM), which was able to suppress the cytoskeletal rearrangements induced by H_2_O_2_ (Fig. 9C). SB203580 alone did not alter the actin cytoskeleton (data not shown).

To explore whether Rho GTPases are involved in the rearrangement of actin fibers, we induced cytoskeletal reorganization with a 3 h exposure to 50 µM Zn^2+^ (Fig. 9D, E, F). Zn^2+^ has been associated with neurodegenerative disorders, glutamate activity and GSH biosynthesis [36], [53]. A half-hour pre-treatment with lysophosphatidic acid (LPA), which is a specific upstream regulator of Rho A, was able to prevent Zn^2+^-induced actin reorganization, indicating that the morphological alterations induced by Zn^2+^ are modulated via the Rho A pathway. No cell death was detected at this concentration (data not shown).

## Discussion

Monotypic CNS cell cultures have significantly contributed to the understanding of brain properties. In this study, we established and characterized a model for cultured astrocytes from adult (90 days old) Wistar rats. We also demonstrate that adult astrocytes respond to oxidative and inflammatory external stimuli. This study is the first to investigate the glutamate metabolism in adult astrocytes *in vitro*. We expect that this methodology may have a significant experimental contribution to the understanding of physiological and pathological functions of astrocytes. This model can provide a useful basis for *in vitro* study of adult astrocyte functions and responses to different types of stimuli. It is important to note the absence of any cells stained positive for neuronal proteins and notably few for microglial protein, which attests to the glial purity of our cultures.

The cultured astrocytes from adult brain could represent an important tool for understanding glial functions because many studies use newborn-derived cells as aging models, despite their plasticity and lability to stimuli. Our cell culture model may have a profile more similar to adult brain, which would be useful for the study of aging. The adult astrocytes demonstrated cellular division to confluence and also possessed important glial properties, such as glutamate uptake, GS activity and GSH content. *In vivo* studies have reported an age-dependent decrease in cerebral glutamate destinations in rats [24], [54], [55], [56], [57]. GSH depletion in glial cells can induce neurotoxicity with impairment of glutamate transporters and increased oxidative stress, which have been described in many neurodegenerative diseases [20], [44], [58].

GFAP and vimentin, two intermediate cytoskeletal filaments, have been identified in astrocytes. The expression of these filaments is tightly regulated during development and is associated with astrocytic differentiation [26], [59]. The exact physiological roles of GFAP and vimentin in astrocytes remain incompletely understood but appear to be involved in cell shape maintenance, CNS cytoarchitecture, mechanical stability and synaptic function [26]. Following CNS injury, the upregulation of both GFAP and vimentin expression displays a major role of reactive astrocytes and contributes to the glial scar [59], [60], [61]. Therefore, it has been suggested that GFAP and vimentin overproduction could be used as specific targets for neuronal repair strategies [62]. Our findings presented in this report demonstrate the presence of GFAP and vimentin in adult astrocytic culture (Fig. 2), which is in agreement with other studies that have demonstrated the coexpression of these proteins in cultured astrocytes [26], [54].

Other astrocytic markers, GS, ALDH1L1, S100B and GAPDH, were also detected. In the CNS, GS is an enzyme expressed only in astrocytes and glutamine synthesis is essential for maintaining neuronal glutamate cycling [63], [64], [65]. Recently, ALDH1L1 was characterized as a new astroglial marker expressed under physiological and pathological conditions [43]. S100B, another astrocytic marker, that is most strongly expressed in the astrocytes, was detected in our cells [66], [67].

Our findings suggest that adult astrocytes are vulnerable to oxidative stimulus, because H_2_O_2_ altered membrane permeability and mitochondrial activity [54]. Similar findings were also reported by Gottfried et al. (2002). As expected, this condition led to a reduction in glutamate uptake and GS activity [43], [57], [68]. Intracellular GSH was most likely decreased due to absence of its precursor glutamate and/or due to the conversion to its oxidized form [69], [70], [71]. Moreover, the metabolic mitochondrial status of adult astrocytes was decreased, as measured by MTT (mainly indicative of succinate-dehydrogenase activity) [54], [72], [73]. Classically, glutamate stimulates glucose uptake in astrocytes [35]. Adult astrocytes are able to take up and utilize glucose, which may be associated with glutamate metabolism and their cytoarchitecture, which enables response to changes in their microenvironment [7], [35], [63].

The effects of resveratrol, a well-known antioxidant compound with a wide range of biological effects, including anti-inflammatory, cardioprotective, neuroprotective and anti-aging activities [45], [74], [75], [76], [77], were also investigated in our adult culture model. Resveratrol was able to prevent H_2_O_2_-induced oxidative stress by modulating antioxidant and inflammatory responses, reinforcing that resveratrol may represent an important strategy for the protection of glial cells [45].

To verify the inflammatory parameters of adult cells further, we measured their production of TNF-α in response to toxins exposure. Santello et al., have recently demonstrated that TNF-α critically controls glutamatergic gliotransmission [46]. Furthermore, TNF-α has other important CNS functions, including injury-mediated microglial and astrocytic activation [47], [78]. Inflammatory stimuli induced a response in adult astrocytes (Fig. 8). Moreover, oxidative stress can also induce an inflammatory response, and TNF-α is essential for the production of other cytokines involved in neuroinflammation, which is another process associated with aging [20].

Under normal conditions, the Rho A signaling pathway induces the formation of stress fibers and focal adhesions [37], [79], [80]. It is known that an increase in intracellular Zn^2+^ concentrations can lead to cell injury [36], and to the best of our knowledge, this report describes the first demonstration that LPA prevents the effect of excess Zn^2+^ on the actin cytoskeleton. The role of p38 MAPK in the astroglial response to oxidative stress was also observed in the cultured adult astrocytes (Fig. 9). Our results demonstrate that extracellular stimuli may exert cytotoxic actions in adult astrocytes *in vitro* via Rho A and MAPK signaling pathway, indicating the importance of the actin cytoskeleton in the maintenance of normal CNS functions.

In summary, the results obtained from adult astrocyte cultures showed the expression of the major astrocytic markers and the functionality of the glutamatergic system and glucose metabolism. These cells may represent an important new tool for *in vitro* and *in vivo* studies because they may faithfully represent the adult brain. Responses to oxidative stress and inflammatory stimuli were also observed, and changes to the cytoskeleton were induced in stimulated astrocytes. Thus, the culture model described in this report elucidates the biochemical and physiological properties of astrocytes and may be useful for understanding the mechanisms involved in the neonatal and adult brain. Furthermore, the biochemical properties of astrocytes from adult Wistar rats submitted to *in vivo* neurotoxicity and neuroprotection studies or experimental models could be ascertained.
